# Death Following Use of Sponge Tents

**Published:** 1874-09

**Authors:** 


*AMERICAN JOURNAL OF OBSTETRICS—August*, 1874.

Death Following the use of Sponge Tents.—Dr. De F. Willard exhibited the uterus of a woman who had died after dilatation of the cervix uteri by sponge tents. * * * He introduced a sponge tent to dilate the cervical canal, which was very narrow. The tent slightly dilated the canal, but as it closed up again, he introduced a larger one, which dilated the canal to the size of the little finger. This gave neuralgic pains, which she bore badly, although a stout-bodied woman. On a Friday he removed this tent, and introduced a smaller one. Contrary to orders the patient worked next day (Saturday) at the sewing machine. On Sunday morning he found her with a tender abdomen, extreme pain, fever, etc. The tent was discovered in the vagina, having slipped out of the canal. The patient was placed upon opiates and supporting treatment, but died on the ninth day. * * *

In the discussion which ensued, Dr. Elwood Wilson reported an analagous case. The patient was desirous of becoming pregnant; she suffered with painful menstruation from malformation of the neck of the uterus. He introduced a sponge tent on a Thursday. Fearing insufficient dilation, he introduced another on Saturday morning. This was left in until Sunday morning. She seemed so well that he gave her permission to go down stairs. She, however, not only did this, but in the evening went to church. In the night she had a chill—on Monday peritonitis set in—on Tuesday she died.

Dr. H. Lennox Hodge also had seen a fatal case. He thought the use of tents was more dangerous than the profession believed. His case differed from the others in this particular, that the patient had been kept perfectly quiet after the introduction of the tents. The dilatation was desired by her husband, himself a physician, to facilitate the diagnosis of a suspected tumor. The first tent was introduced on Saturday; the second and third on Sunday and Monday respectively. Before the removal of the last, she complained of acute abdominal pain, and died of peritonitis in four days after. * * *

Dr. J. L. Ludlow asked whether the danger in the use of the tents was not due to abnormal growths, or to some other abnormal condition of the uterus.

Dr. Hodge replied that in two of the fatal cases mentioned the uteri were healthy, with the exception of a narrowing of the canal of the cervix.

Dr. McCall remarked that a condition of perfect health does not reduce the risk. He related a case in which peritonitis followed the repeated use of the sponge tent; the patient fortunately recovered.

Dr. A. H. Smith remarked that the only fatal case which he could attribute to the use of a tent was one complicated with a morbid growth. In this case he had once dilated the uterus successfully, and removed the tumor with an ecraseur. The tumor recommenced to bleed eighteen months afterwards. He dilated with sponge tents, and scraped away the tumor, which was a soft mass, probably a fibroid degenerating into a medullary sarcoma. Peritonitis set in, and the patient died in three days. He never hesitated to use tents, even in his office. The great danger was from their repeated use, when the uterus is in such an irritable condition that septic matter is readily absorbed. He did not hesitate to use a second tent, but he feared a third. He always required his patients to use an anti-septic wash while the tents were being used. For sterility or for dysmenorrhœa he often put in a sponge tent the day before menstruation, and kept it in throughout the flow.

Dr. Goodell had one case to record of death following the use of three sponge tents. It was a case of intra-mural tumor, and whether the peritonitis was owing to the tents or to the manipulation with finger and sound by the five physicians present, he could not say.

He believed and thought that the history of fatal cases following the use of tents proved that it is not the first tent nor the first batch of tents passed into the cervical canal that does the mischief, but those put in at the second or third visit. The first tent irritates and congests the cervix; its removal abrades the mucous coat, and from this raw surface are absorbed the fetid discharges or septic material generated by the succeeding tents. Influenced by this opinion, he now first stretches open the canal by the uterine dilator, crowds in the largest sponge tent possible, and then insinuates around it several small laminaria tents. He thus tries to accomplish the necessary dilatation by one installment of tents. The use of detergent vaginal washes during the presence of the tents he always enjoins upon his patients.

Dr. J. Cheston Morris stated that his experience led him to agree fully with these views. The first sponge tent was free from danger, and so in a great measure was the second. It was the third introduction that, in his hands, had been followed by serious results.

